# Supplementary material for: Clinical measures of balance and gait cannot differentiate somatosensory impairments in people with lower-limb amputation
Source: Gait Posture. Author manuscript; Available in PMC 2023 Feb 27. (PMC9970031; doi:10.1016/j.gaitpost.2022.10.018)
Supplement: Supplementary Material [file NIHMS1876725-supplement-Supplementary_Material.docx]

Supplementary 1.

# 1.1 Sensation Testing

Light touch and protective (pin prick) sensation were assessed proximally to distally and subjects were instructed to report if they detected a stimulus for light touch or whether a Neurotip examination pin was “sharp” or “dull” for protective sensation. For light touch, a stimulus (finger tip) was applied at target locations for standard neurological assessment of the lower-limb.[1] Stimuli were applied three times per location and sensation was scored out of 2. A score of 0 (indicating no sensation) was recorded if participants were unable to detect a stimulus or correctly identify the stimulus at all, a score of 1 (impaired sensation) if they were able to correctly identify for some trials, and a score of 2 (intact sensation) if they were able to correctly identify the stimulus for all trials. The quality of lower extremity reflexes of the Achilles tendon and the patellar tendon on the intact limb and the patellar tendon (for transtibial amputees) on the residual limb were assessed using a Taylor percussion reflex hammer (hyperreflexive response=3, normal response=2, hyporeflexive response=1, no response=0). Vibration sense was tested using a 128 Hz tuning fork applied perpendicular to the medial malleolus and distal interphalangeal joint of the hallux of the intact limb [2]. For able-bodied controls, both limbs were assessed and the better score was used as a comparison with the AMP group. The tuning fork was struck maximally and participants were asked to report when the sensation started and when they could no longer detect the sensation. The average time between these two points was taken across three trials. Proprioceptive sensation was measured as in standard clinical neurological assessments. The hallux and ankle were moved into a flexed or extended position, using only the sides of the toe or foot to avoid any anteroposterior tactile feedback, and the participant was asked to report either “up” or “down” from the original position. Percent correct out of ten trials was recorded.


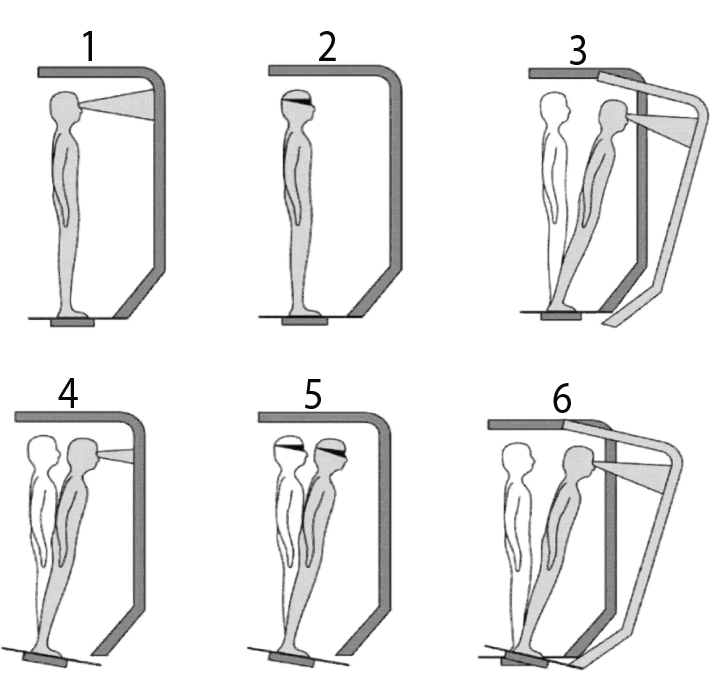


Supplementary Figure 1. Conditions of the Sensory Organization Test (SOT). The SOT is used to measure reliance on visual, vestibular and somatosensory systems for balance. Either visual (eyes closed, surround movement) or somatosensory feedback (platform movement) are altered while measures of sway are assessed across six conditions.

The sensory loss patterns observed in the AMP group are diverse, yet consistent with neuropathy patterns. Typically, the distalmost limbs are most impaired, while the more proximal residual limb is less affected, which may explain why half of our subjects with sensory impairment exhibited impairments only on the intact limb. These findings are also consistent with previous literature reporting sensory loss in this population [3].

# 1.2 Sensory Organization Test

The Sensory Organization Test was implemented using a Neurocom Equitest System, which includes a visual surround that can rotate around the frontal axis and two force plates on a platform that can impart anteroposterior translations and rotate around the frontal axis at the ankles and. During the Sensory Organization Test, the subject is instructed to maintain balance during standing in one of six conditions (Supplementary Figure 1), including (1) stable support surface, eyes open, (2) stable support surface, eyes closed, (3) stable support surface, sway-referenced visual surround, (4) sway-referenced rotating support surface, eyes open, (5) sway-referenced rotating support surface, eyes closed, and (6) sway-referenced visual surround and rotating support surface. Three 20-second trials were completed per condition. Center of pressure (COP) traces were recorded from the force plates (100 Hz), filtered with a low-pass fourth-order Butterworth filter, and analyzed for standard measures of posturography, in addition to clinical measures. Equilibrium scores indicate a participant’s ability to stay within a normative 12.5° anteroposterior sway envelope (Equation 1).

1. $\text{Equilibrium Score=}\frac{{\text{12.5°- (}\text{θ}_{\text{max}}\text{-θ}}_{\text{min}}\text{)}}{\text{12.5°}}$

If a fall was recorded or a full trial was not completed, in accordance with NeuroCom and standard clinical protocol, a zero was recorded for the equilibrium score and the trial was not analyzed for posturography measures. Somatosensory ability (ratio of equilibrium scores in static conditions without vision, condition 2, to equilibrium scores with normal vision, condition 1) indicates a participant’s ability to utilize somatosensation for balance when vision is impaired (Equation 2).

1. $\text{Somatosensory Ability=}\frac{\text{Equilibrium Score}_{\text{condition 2}}}{\text{Equilibrium Score}_{\text{condition 1}}}$

In addition to measures of total body COP, posturography analyses were completed separately for data from the force plate under each of the limbs. Standard posturography measures include excursion (maximum displacement), sway velocity, 95% confidence interval ellipse of sway area, root-mean-square (RMS) distance, and sample entropy in both anteroposterior and mediolateral directions, as described elsewhere [4]. Sample entropy was calculated with a subseries length (m=4), similarity tolerance (r= 0.3), and a time delay (τ=5) according to entropy analyses with posturography data [5]. Additionally, posturography analyses were performed on left and right force plates, separately, to determine potential influence of sensation on each limb’s stability (Supplementary Figure 2). There were no significant differences due to sensory impairment across limbs.


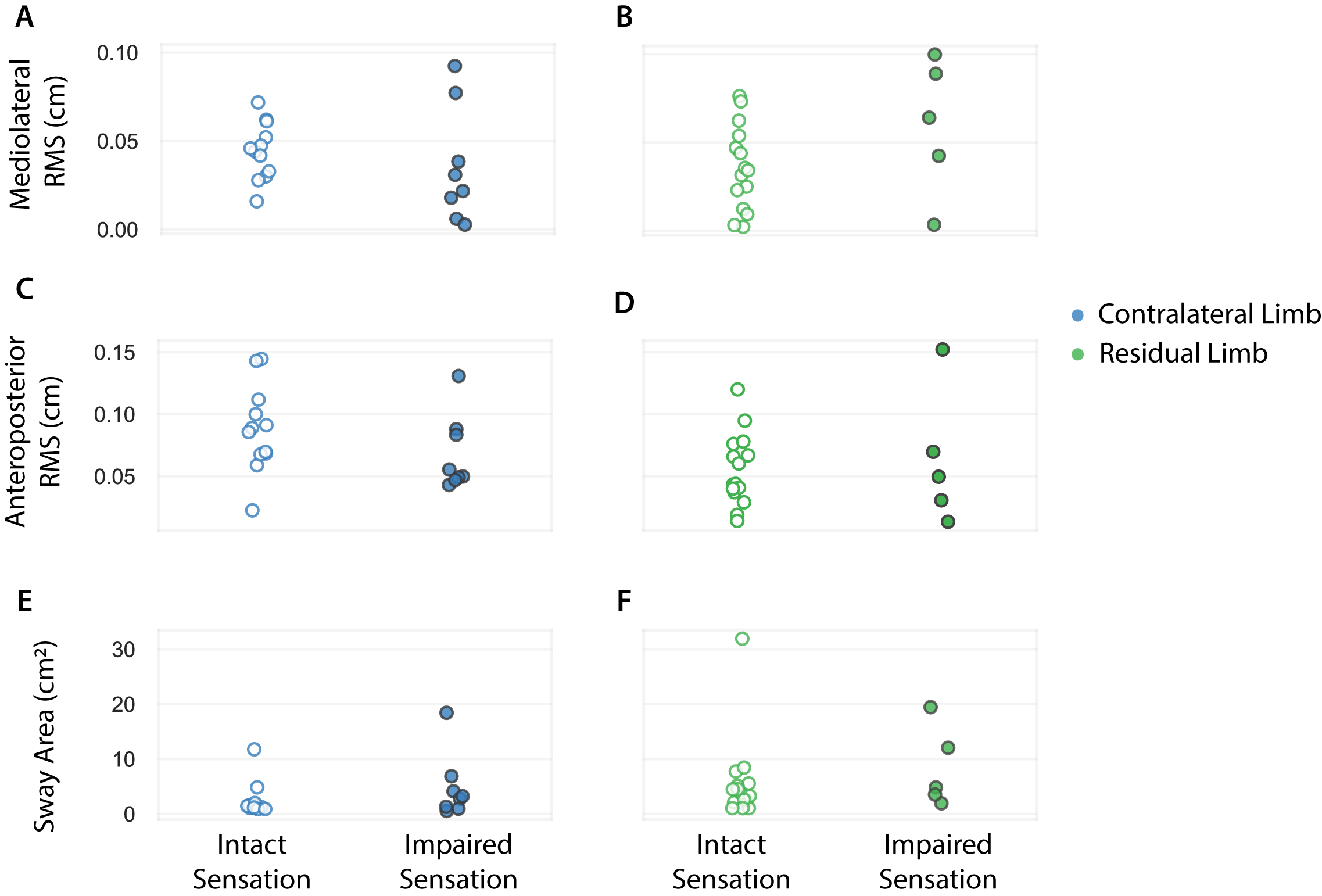


Supplementary Figure 2. Posturography measures for contralateral (blue) and residual limb (green) separated by sensory impairment in that limb. Empty circles indicate intact sensation and filled circles indicate impaired sensation on that limb. (A,B) RMS in mediolateral direction, (C,D) RMS in anteroposterior direction, (E,F) sway area did not significantly differ (p>0.01) when separated by sensory impairment on that limb.

# 1.3 Motor Control Test

The Motor Control Test is a test of reactive balance in which participants’ responses to a translational perturbation are evaluated. The participants were not made aware of the expected motion of this platform, they were instructed only to maintain their balance to the best of their ability. Latency of an active force response after the onset of the perturbation was measured, as well as weight symmetry in stance prior to the perturbation. These are the standard clinical measures of the Equitest system. In individuals with an amputation, the “active response” to the perturbation is too small to detect reliably. Because of this, only the intact limb was used for analysis for latency and the limb with the lower latency was used for able-bodied controls.

# 1.4 Gait Analysis

Gait kinematics during walking on a level surface were recorded using a 16-camera OptiTrack motion analysis system (Flex3 cameras, Natural Point, OR, USA). Six trials were analyzed across a 6-m walkway. Kinematic marker data was collected at 100 Hz and filtered using a 4^th^ order low-pass Butterworth filter at 12 Hz. Step length asymmetry (normalized to stride length, Equation 3), step length variability, and step width variability (standard deviation of step width and coefficient of variation of step width, Equation 4) were calculated as measures of gait stability. Step length was calculated as anteroposterior distance between two consecutive heel markers at heel strike. Step width was calculated as the mediolateral distance between lateral malleolus markers of two consecutive steps. Gait assessments were only collected from 12 of the 20 AMP participants and matched to the corresponding 12 CON participants.

1. $\text{Step Length Asymmetry (SLA)= }\frac{\text{SL}_{\text{intact}}\text{-}\text{SL}_{\text{residual}}}{\text{SL}_{\text{intact}}\text{+}\text{SL}_{\text{residual}}}$
2. $\text{Step}\text{ Width Coefficient of Variation (CV)}\text{= }\frac{\text{Standard }\text{Devation}\text{ (SD) Step Width}}{\text{Mean Step Width}}*100$

# 1.5 Statistical Analysis

Though there are only four individuals with a transfemoral amputation, these individuals were not outliers in our data. Furthermore, when used as a co-variate in a linear regression model, the relationship between level of amputation and functional measures was not significant. Thus, these individuals were included in our analysis with the rest of the AMP group. Supplementary Tables 1-4 depict any significant differences between groups for both CON and AMP groups and, within the AMP group, between those with full sensation and impaired sensation. Significance level was a priori set at 0.05, with Bonferroni corrections completed for each group of comparisons.

|  | CON | AMP | p | Full Sensation | Impaired Sensation | p |
| --- | --- | --- | --- | --- | --- | --- |
| Condition 1: Eyes open, static platform | 94.14+1.75 | 92.62+1.97 | 0.011 | 93.21+1.96 | 92.04+1.90 | 0.082 |
| Condition 2: Eyes closed, static platform | 89.73+ 3.69 | 83.16+8.67 | 0.0017* | 84.79+5.11 | 81.53+11.25 | 0.82 |
| Condition 3: Surround sway, static platform | 88.74+2.77 | 80.70+12.16 | 6.3e-5* | 83.61+4.51 | 77.78+16.52 | 0.36 |
| Condition 4: Eyes open, platform sway | 75.87+10.51 | 83.91+6.42 | 0.021 | 85.05+3.90 | 82.77+8.31 | 0.94 |
| Condition 5: Eyes closed, platform sway | 50.84+18.88 | 48.52+20.93 | 0.96 | 51.61+18.90 | 45.44+23.38 | 0.65 |
| Condition 6: Surround and platform sway | 50.83+16.12 | 48.06+25.90 | 0.89 | 55.19+20.20 | 40.94+29.93 | 0.26 |
| Composite Score | 70.19+8.16 | 68.10+11.50 | 0.76 | 71.74+7.53 | 64.46+13.89 | 0.26 |
| Somatosensory Ability | 0.95+0.03 | 0.90+0.08 | 0.0031* | 0.91+0.042 | 0.88+0.11 | 0.9 |

Supplementary Table 1. Clinical measures of Sensory Organization Test. Mean ± standard deviation of equilibrium scores for all conditions, as well as composite equilibrium score and somatosensory ability for AMP group based on sensation (full sensation vs. impaired sensation) and for control (CON) vs amputation (AMP) groups. (* indicates statistically significant difference, p<0.007)

|  | CON | AMP | p | Full Sensation | Impaired Sensation | p |
| --- | --- | --- | --- | --- | --- | --- |
| Latency (ms) | 131.56+12.87 | 150.16+18.19 | 0.0021* | 145.36+15.30 | 153.89+20.24 | 0.79 |
| Symmetry (%) | -0.20+4.32 | -16.546+13.68 | 0.0013* | -14.43+18.09 | -18.19+9.93 | 0.27 |

Supplementary Table 3. Measures of reactive balance for the Motor Control Test. Mean + standard deviation of latency of responses (on the intact limb only) and weight-bearing symmetry prior to responses for AMP group based on sensation (full sensation vs. impaired sensation) and for control (CON) vs amputation (AMP) groups. (* indicates statistically significant difference, p<0.025)

|  | CON | AMP | p | Full Sensation | Impaired Sensation | p |
| --- | --- | --- | --- | --- | --- | --- |
| Excursion ML (cm) | 0.81+0.39 | 1.51+0.97 | 0.0032* | 1.60+1.26 | 1.41+0.62 | 0.82 |
| Sway velocity (cm/s) | 0.81+0.26 | 1.47+0.42 | 3.8e-6* | 1.39+0.32 | 1.55+0.51 | 0.60 |
| Sway Area (cm^2^) | 5.98+1.33 | 2.33+4.51 | .00021* | 5.58+4.58 | 6.37+4.65 | 0.50 |
| RMS Distance AP | 0.047+0.035 | 0.047+0.034 | 0.93 | 0.051+0.037 | 0.042+0.034 | 0.82 |
| RMS Distance ML | 0.013+0.0095 | .046+0.025 | 3.8e-6* | 0.040+0.023 | 0.053+0.025 | 0.23 |
| Sample Entropy AP | 0.26+0.093 | 0.34+ 0.075 | 0.0073 | 0.34+0.084 | 0.34+0.069 | 0.71 |
| Sample Entropy ML | 0.26+0.088 | 0.29+0.069 | 0.26 | 0.30+0.090 | 0.29+0.046 | 1.0 |
| Change in Area  (Eyes open to eyes closed, cm^2^) | 3.03+3.31 | 16.05+19.77 | 0.00071* | 12.83+8.85 | 19.27+26.90 | 0.76 |

Supplementary Table 2. Posturography measures of static balance with eyes open condition and change in area (eyes open to eyes closed). Mean + standard deviation of posturography measures for AMP group based on sensation (full sensation vs. impaired sensation) and for control (CON) vs amputation (AMP) groups. (* indicates statistically significant difference, p<0.00625)

|  | CON | AMP | p | Full Sensation | Impaired Sensation | p |
| --- | --- | --- | --- | --- | --- | --- |
| Functional Gait Assessment Score | 28.6+1.9 | 19.0+5.0 | 1.9e-06* | 20.7+4.1 | 17.2+5.4 | 0.12 |
| Step Length Asymmetry (au) | -0.0041+0.048 | -0.0070+0.037 | 0.91 | -0.0059+0.032 | -0.0082+0.045 | 0.94 |
| Step Width Variability (CV) | 33.26+14.35 | 29.03+14.14 | 0.52 | 35.61+14.81 | 22.46+10.80 | 0.13 |
| Step Width Variability (SD) | 3.30+1.17 | 3.74+1.20 | 0.42 | 4.15+1.45 | 3.32+0.82 | 0.24 |

Supplementary Table 4. Clinical and biomechanical measures of gait for both controls (CON) vs amputation (AMP) groups and full vs impaired sensation within the AMP group. (* indicates statistically significant difference, p<0.0125)

# 1.6 Bibliography

[1] F.M. Maynard, M.B. Bracken, G. Creasey, W.H. Donovan, T.B. Ducker, S.L. Garber, R.J. Marino, S.L. Stover, C.H. Tator, R.L. Waters, International standards for neurological and functional classification of spinal cord injury, Spinal Cord. 35 (1997) 266–274.

[2] A.T. Prabhakar, T. Suresh, D.S. Kurian, V. Mathew, A.I.A. Shaik, S. Aaron, A. Sivadasan, R.N. Benjamin, M. Alexander, Timed Vibration Sense and Joint Position Sense Testing in the Diagnosis of Distal Sensory Polyneuropathy., J. Neurosci. Rural Pract. 10 (2019) 273–277. <https://doi.org/10.4103/jnrp.jnrp_241_18>.

[3] C.A. Templeton, N.D.J. Strzalkowski, P. Galvin, L.R. Bent, Cutaneous sensitivity in unilateral trans-tibial amputees, PLoS One. 13 (2018) e0197557. https://doi.org/10.1371/journal.pone.0197557.

[4] T.E. Prieto, J.B. Myklebust, R.G. Hoffmann, E.G. Lovett, B.M. Myklebust, Measures of postural steadiness: differences between healthy young and elderly adults, IEEE Trans. Biomed. Eng. 43 (1996) 956–966. https://doi.org/10.1109/10.532130.

[5] L. Montesinos, R. Castaldo, L. Pecchia, On the use of approximate entropy and sample entropy with centre of pressure time-series, J. Neuroeng. Rehabil. 15 (2018) 116. https://doi.org/10.1186/s12984-018-0465-9.
